# A multimodal approach to equine thoracolumbar myofascial pain: integrating clinical examination, AI-based gait analysis, and the ridden horse pain ethogram

**DOI:** 10.1186/s12917-026-05566-w

**Published:** 2026-06-09

**Authors:** María Resano-Zuazu, Jorge U. Carmona, David Argüelles

**Affiliations:** 1 Department of Animal Medicine and Surgery, Faculty of Veterinary Medicine, Universidad de Córdoba, Campus de Rabanales, Córdoba, 14071 Spain; 2 Departamento de Salud Animal, Grupo de Investigación Terapia Regenerativa, Universidad de Caldas, Calle 65 No 26-10, Manizales, 170004 Colombia

**Keywords:** Trigger points, Palpation, Back pain, Rider, Biomechanics, Machine learning

## Abstract

**Background:**

Myofascial pain syndrome (MPS) is considered highly prevalent in horses, yet it is frequently underdiagnosed due to limited understanding of its clinical presentation and functional consequences, as well as the inherent challenges associated with its clinical assessment and objective measurement. In equines, the relationship between palpation-based MPS indicators, gait asymmetries, and pain-related behaviours during ridden work remains unclear. The primary aim of this study was to evaluate the association between thoracolumbar palpation pain scores and gait asymmetries measured in-hand and during ridden exercise, as well as behavioural indicators of pain assessed using the ridden horse pain ethogram (RHpE). The secondary aim was to explore the effect of a rider on gait asymmetry using a within-horse comparison between in-hand and ridden conditions. Clinical and behavioural signs and demographic variables were also described.

**Results:**

Twenty police working horses from the Special Mounted Unit of the Spanish National Police in Madrid were included. At least one myofascial trigger point, identified by a palpable taut band and a hypersensitive spot, was identified in all horses (100%) in the thoracolumbar region, followed by the jump sign (85%) and restricted range of motion (80%). Local twitch responses were not observed in any horse (0%). Clinical indicators of MPS were not associated with objective gait asymmetries measures in either condition. Forelimb push-off asymmetry (HDmax) increased under ridden conditions (β = 0.196, 95% CI 0.028–0.034, *p =* 0.022). Training level modified this effect, with higher-level horses exhibiting greater increases in HDmax when ridden. The mean RHpE score was 7.2, with 40% of horses scoring ≥ 8 behaviours. The most frequent behaviours were ears rotated back (95%), mouth opening (95%), and intense stare (80%). No significant associations were found between RHpE scores and either MPS clinical signs or ridden gait asymmetries.

**Conclusions:**

This is the first study integrating palpation-based assessment of thoracolumbar MPS with objective gait analysis and the RHpE. The findings suggest the importance of palpation as the primary diagnostic approach for MPS in horses and indicate that a multimodal approach may improve the understanding and management of equine MPS.

**Supplementary Information:**

The online version contains supplementary material available at 10.1186/s12917-026-05566-w.

## Background

Myofascial pain syndrome (MPS) in back muscles is increasingly recognised as a contributor to poor performance in horses [1–3]. Recent studies have identified the *longissimus thoracis* as the muscle with the highest prevalence of trigger points (TrPs), affecting 71% of dressage horses and 86% of show-jumping horses [1]. MPS, characterised by the presence of TrPs, is the most common manifestation of chronic pain and is commonly thought to be associated with low-grade lameness [3, 4]. Lameness associated with MPS cannot be localised through conventional diagnostic methods, including imaging or nerve blocks [3, 4]. According to human literature, MPS may occur as a primary condition, independent of other pain generators. However, it frequently coexists with, or is secondary to, other acute and chronic painful musculoskeletal conditions [5].

Despite its clinical relevance, the diagnosis of equine back MPS remains challenging due to the limited scientific literature [1, 2, 4, 6], insufficient understanding of its clinical presentation and functional consequences, and the absence of reliable and valid measurements methods. Physical examination, primarily based on manual palpation of the muscle, has primarily been used for back MPS diagnosis in humans [7] and horses [1, 2]. Classical diagnostic criteria in humans include tenderness in a taut band, a referred pain pattern, pain recognition, and the local twitch response (LTR), defined as a transient contraction of the palpable taut band [8], limited range of motion (ROM), the jump sign, defined as a withdrawal reaction to palpation based on behavioural responses [8], and muscle weakness without atrophy [9]. Recent research has updated the diagnostic criteria for MPS [10, 11]. Current recommendations suggest that at least two of the following features should be present for diagnosis: a taut band, a hypersensitive spot, and referred pain [11]. LTR has also been highlighted as a key criterion [10]. No specific diagnostic criteria for equine MPS have been established; the signs currently described in the pectoral and brachiocephalicus muscles are extrapolated from human studies [12, 13]. These include the presence of a taut band, a jump sign, and an LTR. Specific criteria for the diagnosis of equine back MPS have not yet been established.

In humans, facial expressions and body language have influenced examiners when diagnosing MPS in the upper quarter muscles, even though these factors were not part of the established diagnostic criteria for MPS [14]. In horses, facial expressions and behavioural indicators play an important role in the assessment of musculoskeletal pain, as they are non-verbal animals [15]. Behaviours attributed to pain during palpation of TrPs have been described in the equine brachiocephalicus and pectoral muscles [12, 13]. The ridden horse pain ethogram (RHpE) has been developed to facilitate the recognition of musculoskeletal pain in ridden horses [16]. It characterises specific facial expressions and behaviours associated with pain or stress [16–18]. The RHpE comprises 24 behaviours, most of which are displayed at least ten times more frequently in lame horses compared to non-lame horses [16]. A significant reduction in RHpE scores following the elimination of lameness through diagnostic anaesthesia provides strong evidence of a causal association between these behaviours and pain [19, 20]. The presence of eight or more RHpE behaviours is considered to be associated with the expression of musculoskeletal pain-related behaviour [16, 19–21]. No studies have established a correlation between MPS and its specific diagnostic criteria in the equine thoracolumbar region and the RHpE.

Gait analysis has become a valuable tool for quantifying movement asymmetries associated with pain or dysfunction in horses, with emerging artificial intelligence (AI)-based approaches showing increasing potential for clinical application [22–25]. Gait changes have been observed in humans with non-specific low back pain, suggesting that MPS may be associated with changes in gait parameters [26, 27]. Previous studies using kinematic analysis have demonstrated altered back movement patterns in horses with back dysfunction compared to sound horses [28, 29]. In veterinary medicine, there are no studies investigating the biomechanical implications of MPS in horses using gait analysis. To date, only one report has investigated the short-term biomechanical effects of dry needling technique for MPS using AI-based gait analysis [30].

The primary aim of this study was to evaluate the association between thoracolumbar MPS, assessed by palpation, and locomotor and behavioural outcomes in horses. Specifically, the relationship between palpation pain score and (1) gait asymmetries measured in-hand, (2) gait asymmetries measured during ridden exercise, and (3) behavioural indicators of musculoskeletal pain using the RHpE was analysed. As a secondary aim, the potential effect of the presence of a rider on objective gait asymmetry was explored using within-horse comparisons between in-hand and ridden conditions. Additionally, the study aimed to (4) describe the presence of common clinical signs of MPS, (5) describe behavioural signs assessed using the RHpE, and (6) explore the influence of demographic variables (sex, age, discipline, and training level) within the statistical analysis. It was hypothesised that horses with higher thoracolumbar pain scores would exhibit greater gait asymmetries and higher RHpE scores.

## Methods

**Fig. 1:**
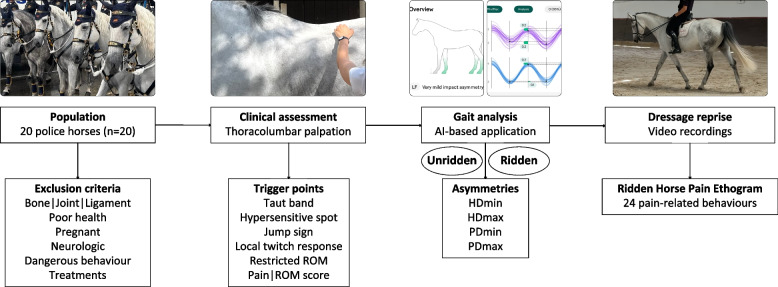
Design of the study

This study was designed as a cross-sectional observational with correlational analysis. The study design is summarised in Fig. 1. According to the Ethics Committee of the University of Córdoba, no specific ethical approval was required, as the project involved routine professional procedures that fall outside the scope of current legislation (Real Decreto 53/2013, of February 1 st, which establishes the basic standards for the protection of animals used in experimentation and other scientific purposes, including teaching*.* Official publication: Boletín Oficial del Estado (B.O.E); number: 34/2013; publication date: 8 February 2013; page number: 11370–11421). All interventions were performed in accordance with the Code of Good Veterinary Practice [31]. The study was conducted under a formal collaboration agreement and received written authorization from the National Police Special Units Headquarters (Document Registration Number: S2025005500500000982).

### Horse population

A population of 20 horses of different ages, sexes, breeds, and disciplines from the Special Mounted Unit of the Spanish National Police in Madrid participated in this study. Horses were recruited using convenience sampling according to on-site availability. Individual data for each horse are provided in Additional file 1. All animals were housed in stables under similar conditions and followed similar management routines. They received the same type of feed, and their daily routine involved work either in the training arenas or on patrols in the city. Their performance level in ridden work was basic, intermediate, or advanced.

No a priori sample size calculation was performed, as this study was conducted under field-based clinical conditions and the number of horses was determined by operational availability within the police unit. To provide context for the inferential scope of the sample, a post hoc sensitivity analysis was undertaken for the primary within horse-comparison between unridden and ridden conditions. Based on paired design with a sample size of 20 horses and a two-sided significance level of 0.05, the study was able to detect moderate-to-large effect sizes. In the present dataset, the observed effect size for the main outcome (forelimb push-off asymmetry, HDmax) was moderate (Cohen’s d ≈ 0.65), supporting that the study was adequately sensitive to detect effects of this magnitude. Smaller effects may not have been detectable and should be interpreted with caution.

Horses routinely engaged in ridden work and in good physical condition to perform a working session at walk, trot, and canter were included. Horses were excluded if they had received analgesic or anti-inflammatory medication within the previous 48 h, had a previous diagnosis of bone, joint, or ligament pathology, had a diagnosis of neurological disorders, presented poor health, were pregnant mares, exhibited behaviour incompatible with safe clinical assessment or handling, were dangerous to handle under saddle, had undergone physiotherapy treatments within the previous week, or showed signs of lameness on the day of evaluation.

### Thoracolumbar clinical assessment

All horses were examined while standing squarely on a firm and level surface outside the stable [32]. Each animal was fitted with a halter and a lead rope, which was loosely tied to allow the horse to express natural behavioural reactions. Each assessment lasted approximately fifteen minutes per animal. All assessments were performed by a single experienced examiner.

The epaxial muscles were evaluated bilaterally, beginning on the left side and then on the right. Palpation was initiated at the base of the withers and continued caudally towards the lumbosacral junction [33]. The assessment area included the region approximately 10 cm lateral to the spinous processes on both sides of the spine. The epaxial region was assessed as a single anatomical unit. A preliminary contact was performed using the flat of the hand and relaxed fingers [34] to habituate the horse to the touch of the examiner and promote relaxation during the procedure. Both hands were placed side by side over the examined area to ensure similar pressure distribution.

#### Palpation assessment for TrPs

Palpation was conducted to identify the following features of myofascial TrPs: a palpable taut band, a hypersensitive spot, a jump sign, restricted ROM, and an LTR. Findings were graded and recorded on a present/absent basis. To identify a taut band, palpation was performed using a light firm touch [32], perpendicular to the muscle fibers [7]. Once the taut band was identified, direct pressure was applied to confirm the presence of a myofascial TrP [12].

Responses to palpation were graded on a 0–5 scale according to muscle tone and behavioural reaction [35]: soft and low tone (0), normal (1), increased muscle tone but not painful (2), increased muscle tone and/or painful (3), painful (4), and very painful (5) (Additional file 2. Table 1). A single pain score was assigned per horse based on the response to palpation of these findings.

#### ROM assessment

In addition, a mobility test was performed once per horse during static clinical assessment to assess thoracolumbar ROM, including flexion, extension, and lateral flexion with rotation. Flexion was induced through manual stimulation applied along the ventral midline, which promoted lifting of the cranial thoracic area [36, 37]. To evaluate extension, bilateral digital stimulation in the form of tickling was applied at the base of the withers and over the T16-T18 region [38]. Lateral flexion combined with rotation was evaluated by gently guiding the base of the tail toward the examiner while simultaneously applying pressure against the body of the horse at the level of the last ribs [38]. Mobility was scored 0–4: hypermobility (0), normal (1), mild (2), moderate (3), and severe (4) [39] (Additional file 2. Table 2). A single overall score was assigned for each evaluation, as the ROM outcome was intended as a global functional assessment. For analysis, ROM restriction was recorded as present if limitation was observed in any direction.

### Gait analysis

**Fig. 2:**
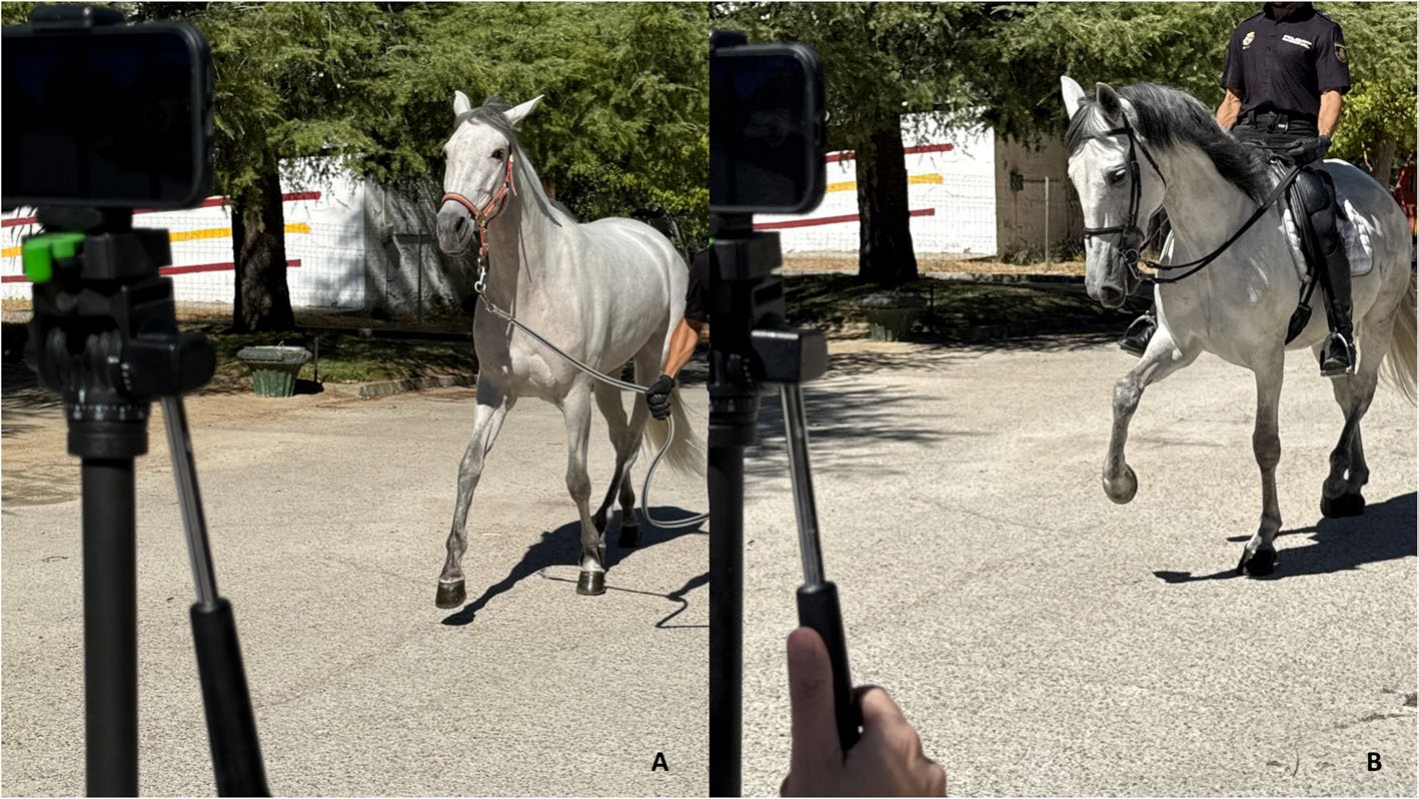
Artificial intelligence (AI)-based gait analysis under **A**) unridden and **B**) ridden conditions

All horses were filmed outdoors in the same location, under natural lighting conditions, on level and even ground. All the horses were led by the same handler. The gait assessment was conducted sequentially: first in-hand (without a rider) and subsequently under saddle (with tack and rider) (Fig. 2). In both situations, horses were trotted along a 30 m straight line on a hard surface, twice back and forth. During the in-hand trials, horses were equipped with a head collar and a loose lead line to allow free head movement and were trotted at a comfortable and steady speed. For the ridden assessment, all horses were equipped with the same model of tack, consisting of a St George model dressage saddle with knee rolls (Zaldi Sillas de Montar S.A., Salamanca, Spain) and a rubber Pelham bit (SEFTON, Hispano Hípica S.A., Salamanca, Spain). The rider was instructed to keep the reins loose and maintain a sitting trot through the recordings.

Biomechanical data were collected using an iPhone 16 Pro (iOS version 18.5, Apple Inc., Cupertino, CA, USA) equipped with the Sleip mobile phone application (app) (version 5.11.0.3, Sleip AI AB, Stockholm, Sweden). The iPhone was positioned approximately 1.5 m above the ground on a professional tripod (Cullmann 2108 model, Transcontinenta GmbH, Fürth, Germany) placed in front of the horse. Recordings were made in a landscape orientation at eye level. Video recordings were first saved on the mobile phone and later uploaded for data processing. Data acquisition for all horses was manually initiated and concluded by the same operator. Asymmetry data for both the head and the pelvis were calculated by the app according to anatomical scaling based on the size of the horse [40].

Severity of the asymmetries was defined and categorized as follows: 0.0–0.2, no asymmetry (0); 0.3–0.5, very mild asymmetry (1); 0.6–0.9, mild asymmetry (2); 1.0–1.4, moderate asymmetry (3); 1.5–2.0, severe asymmetry (4) [22].

### Ethogram-based equine pain assessment

All horses were ridden by the same professional and experienced rider throughout the study. A purpose-designed dressage-type test was implemented, including exercises at walk, trot and canter on both reins. The detailed dressage test is provided (Additional file 3). The test was designed by the experienced rider. A common baseline structure was used; however, the level of exercises was adapted to each horse performance capacity, as determined by the experienced rider, in order to capture the full repertoire of movements, in accordance with published recommendations [41]. The basic level included walk, trot, canter, and leg-yield exercises. The intermediate level comprised all of the above, plus lateral movements such as shoulder-in, haunches-in and half-pass. The advanced level included the previous exercises as well as high-school movements such as piaffe, passage, and flying changes. All trot-work was performed in sitting trot. Tests were performed in a 40 × 20 m indoor arena with a sand surface, and the duration of each test was designed to be between 5 and 10 min. Models of tack and recording equipment were identical to those used for gait analysis.

Video recordings of all tests were acquired under natural light from a fixed position in one corner of the arena, between M and C, using the mobile phone on the tripod in landscape orientation. Horses were warmed up for 5 min prior to the test in the arena. Each horse was recorded from behind, from both sides, and from the front, on both reins. The visibility of the sclera of the horse at rest was assessed before each test. During the ridden work, the rider had no knowledge of the palpation assessment results. To avoid recall bias, the RHpE scoring was applied retrospectively by a single assessor to the video recordings one month later. A complete description of the 24 behaviours types included in the RHpE is presented [41] (Additional file 4).

### Intervention

Physical assessments, gait analysis recordings, ridden test recordings, and RHpE scoring assessments were conducted by a single veterinarian (M.R.Z.) specialised in equine rehabilitation, with clinical experience in the assessment of MPS and specific training in TrP palpation, as well as in both the Sleip app and the RHpE [41]. Handling of the horses, dressage-test design, and ridden work were performed by the same individual (F.A.A.), who acted as both handler and rider for all the horses to ensure standardisation of the procedure. F.A.A. is a lead rider of the Special Mounted Unit, specialises in the rehabilitation of service horses using classical training principles, and has a background in veterinary medicine. 

### Statistical analysis

All statistical analyses were performed in R (version 4.5.2; R Foundation for Statistical Computing, Vienna, Austria). An exploratory analytical approach was used to evaluate the effect of rider condition on objective gait asymmetries and to investigate their relationship with clinical findings.

Biomechanical outcomes were derived from the Sleip system and included continuous measures of head and hindlimb asymmetry during impact (HDmin, PDmin) and push-off (HDmax, PDmax) phases. Absolute values were analysed, because the direction of asymmetry was not considered relevant to the study objectives. Values closer to zero were interpreted as reflecting greater symmetry.

Each horse was evaluated under two consecutive conditions, in-hand (unridden) and ridden, resulting in a within-horse repeated-measures design. Accordingly, rider condition was included as the main explanatory variable in all analyses. Base models were initially fitted as Gaussian generalized linear mixed-effects models (GLMMs) with identity link, including horse identity as a random intercept to account for within-horse correlation. When the estimated between-horse variance was negligible or not estimable, models were simplified to linear models (LM) to avoid overparameterisation and preserve model stability. All models were adjusted for sex, age, and body weight.

The potential modifying effect of training level was evaluated by including an interaction term with rider condition. Clinical variables (pain score, jump sign, and thoracolumbar ROM) were explored individually as fixed effects to assess their association with gait asymmetries and their potential influence on the rider-condition effect. Variables that did not contribute meaningfully to model fit or interpretation were not retained in the final models.

Analyses involving the RHpE were restricted to the ridden condition (one observation per horse). Associations between RHpE score and clinical variables were explored using Spearman’s rank correlation coefficient, with p-values adjusted for multiple comparisons using the false discovery rate (Benjamini–Hochberg method). Additional exploratory linear regression models were fitted to evaluate whether biomechanical asymmetries or clinical findings explained variation in RHpE score.

Given the exploratory nature of the study, results were interpreted primarily on the basis of effect size, consistency, and biological plausibility. A two-sided significance level of *p* < 0.05 was considered for descriptive purposes.

## Results

### Demographic profile of the equine population

The study population consisted of 20 horses, including 6 geldings (30%) and 14 mares (70%), with a median age of 10.85 (SD 4.59, range 5–18) years and a median body weight of 514.1 (SD 34.83, range 458–589) kg. The breeds represented were 4 warmbloods, specifically Spanish Sport Horses (20%), 12 Pure Spanish Horses (60%), and 4 crossbreeds (20%). Regarding their aptitude, 16 horses were used for dressage (80%) and four for show jumping (20%). In terms of training level, 15 were classified as basic (75%), 2 as intermediate (10%), and 3 as advanced (15%).

### MPS indicators

At least one myofascial TrP, identified by a palpable taut band and a hypersensitive spot, was identified in all horses (100%). LTRs were not observed in any horse (0%). Pain on palpation was present in all horses (100%), with a mean pain score of 4.45 (SD 0.69; range 3–5). Jump sign was identified in 85% of horses, while restricted thoracolumbar ROM was present in 80%, with a mean ROM score of 2.32 (SD 0.73).

### Objective gait asymmetries: unridden vs ridden

Objective gait asymmetry measures were evaluated under unridden and ridden conditions. Individual asymmetry values for each horse in both conditions are provided in the supplementary material (Additional file 5). Among the variables analysed, HDmax differed between conditions (*p =* 0.022), with higher values observed in the ridden condition compared with the unridden condition (Table 1).

**Table 1:** Fixed-effects estimates for the effect of rider (unridden vs ridden) on absolute asymmetry measures

Outcome	Model	Estimate (β)	95% CI	*p*-value
HDmin	GLMM	0.117	− 0.085 to 0.319	0.255
PDmin	LM	0.001	− 0.115 to 0.117	0.990
HDmax	LM	0.196	0.028 to 0.364	0.022
PDmax	LM	− 0.111	− 0.289 to 0.067	0.235

Estimates represent the adjusted mean difference between ridden and unridden conditions. Positive values indicate increased asymmetry in the ridden condition. Models were adjusted for sex, age, body weight, and training level. A random intercept for horse was included when supported by between-horse variance (HDmin). Confidence intervals correspond to Wald 95% intervals (β ± 1.96·SE)

*HDmin* Head impact mean, *HDmax* Head push-off mean, *PDmin* Hind impact mean, *PDmax* hind push-off mean, *LM* Linear model, *GLMM* Generalized linear mixed-effects model, *CI* Confidence interval

No differences between conditions were detected for HDmin (*p =* 0.255), PDmin (*p =* 0.990), or PDmax (*p =* 0.235). Condition-specific asymmetry estimates are presented in Table 2.

**Table 2:** Adjusted asymmetry estimates by rider condition

Outcome	Condition	Estimate (95% CI)
HDmin	In hand	0.338 (0.156–0.520)
HDmin	Ridden	0.540 (0.358–0.722)
PDmin	In hand	0.275 (0.196–0.354)
PDmin	Ridden	0.250 (0.171–0.328)
HDmax	In hand	0.205 (0.086–0.324)
HDmax	Ridden	0.524 (0.406–0.643)
PDmax	In hand	0.333 (0.204–0.462)
PDmax	Ridden	0.253 (0.124–0.382)

Values are estimated marginal means (EMMs) with 95% confidence intervals derived from models adjusted for sex, age, and body weight. HDmin was analysed using a Gaussian generalized linear mixed-effects model including horse identity as a random intercept. For PDmin, HDmax, and PDmax, linear models were used, as the between-horse variance component was negligible or not estimable. Absolute asymmetry values were analysed. Abbreviations as in Table 1

#### Effect of training level on ridden gait asymmetries

**Fig. 3:**
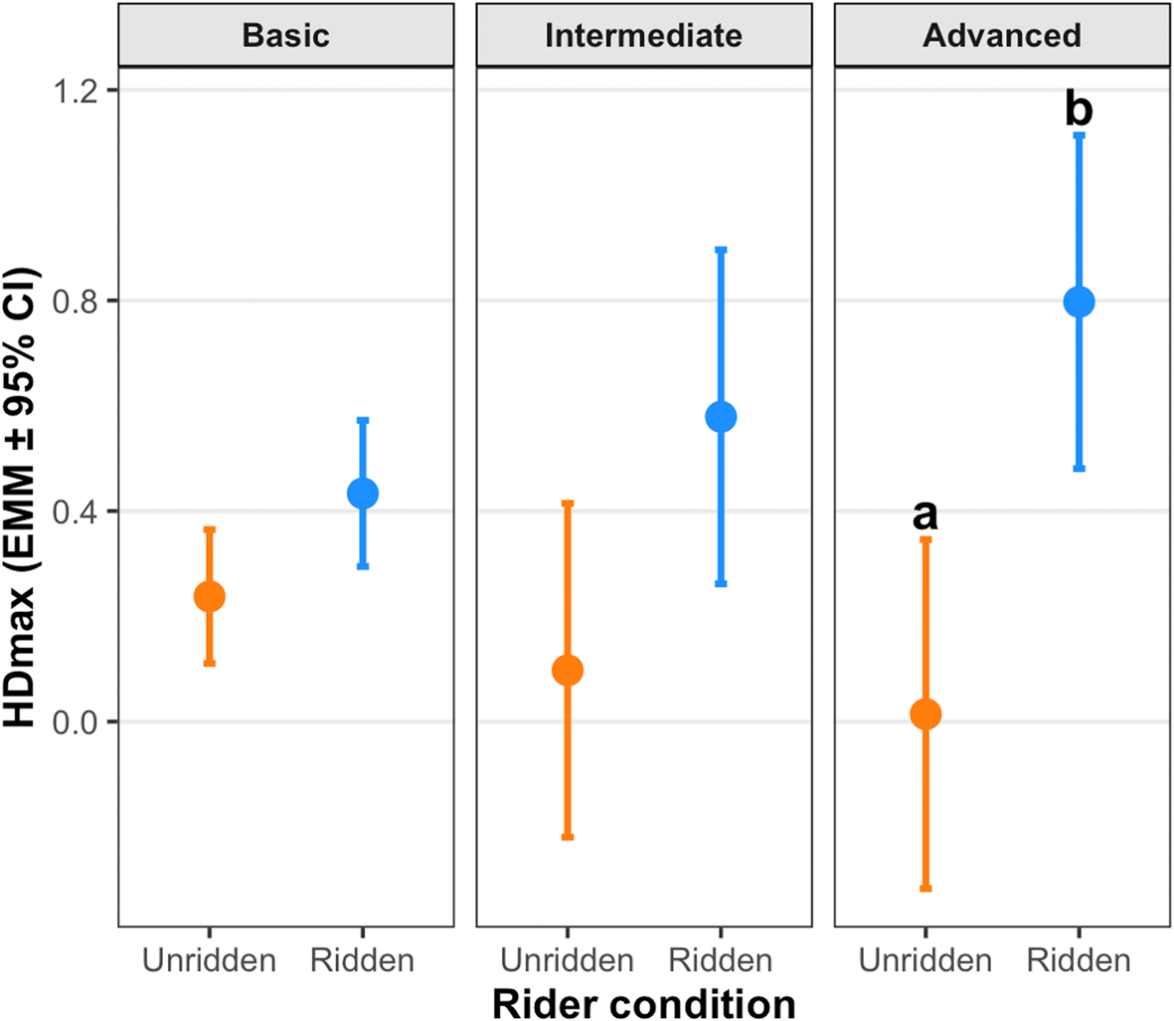
Estimated marginal means (± 95% confidence intervals) of head push-off mean (HDmax) under unridden and ridden conditions, stratified by training level (Basic, Intermediate, and Advanced). Estimates were adjusted for sex, age, and body weight. Higher training levels showed a greater increase in asymmetry magnitude under the ridden condition. Different lowercase letters indicate statistically significant differences between rider conditions within the same training level according to Holm-adjusted pairwise comparisons; groups sharing the same letter do not differ significantly

Training level was associated with differences in HDmax under the ridden condition. An interaction between rider condition and training level was observed for HDmax, with horses at higher training levels showing a greater increase in asymmetry magnitude when ridden (Fig. 3).

Impact-related measures (HDmin and PDmin) did not demonstrate consistent differences across training levels, and effect estimates remained small relative to within-group variability. PDmax followed a similar but less pronounced pattern. Model coefficients for the interaction between rider condition and training level are summarised in Table 3. Given the limited number of horses in the intermediate and advanced training categories, these findings are presented descriptively.

**Table 3:** Fixed-effects estimates for the interaction between rider and training level on absolute asymmetry measures

Outcome (absolute)	Model	Training level	Estimate (β)	95% CI	*p*-value
HDmin	GLMM	Intermediate vs basic	0.041	− 0.214 to 0.296	0.753
		Advanced vs basic	0.089	− 0.182 to 0.360	0.514
PDmin	LM	Intermediate vs basic	− 0.018	− 0.149 to 0.113	0.787
		Advanced vs basic	0.027	− 0.128 to 0.182	0.726
HDmax	LM	Intermediate vs basic	0.112	− 0.071 to 0.295	0.229
		Advanced vs basic	0.284	0.031 to 0.537	0.029
PDmax	LM	Intermediate vs basic	− 0.064	− 0.219 to 0.091	0.412
		Advanced vs basic	0.118	− 0.084 to 0.320	0.245

Estimates represent the additional change in asymmetry magnitude in the ridden condition relative to the basic training level. Positive values indicate greater asymmetry under ridden conditions compared with the basic level. Models were adjusted for sex, age, body weight, and training level. A random intercept for horse was included when supported by between-horse variance (HDmin). Due to the limited number of horses in the intermediate and advanced training categories, estimates are presented descriptively. Confidence intervals correspond to Wald 95% intervals. IC95% calculated as Wald intervals (β ± 1.96·SE). Acronyms as in Table 1

#### Clinical findings and asymmetries: absence of association

**Fig. 4:**
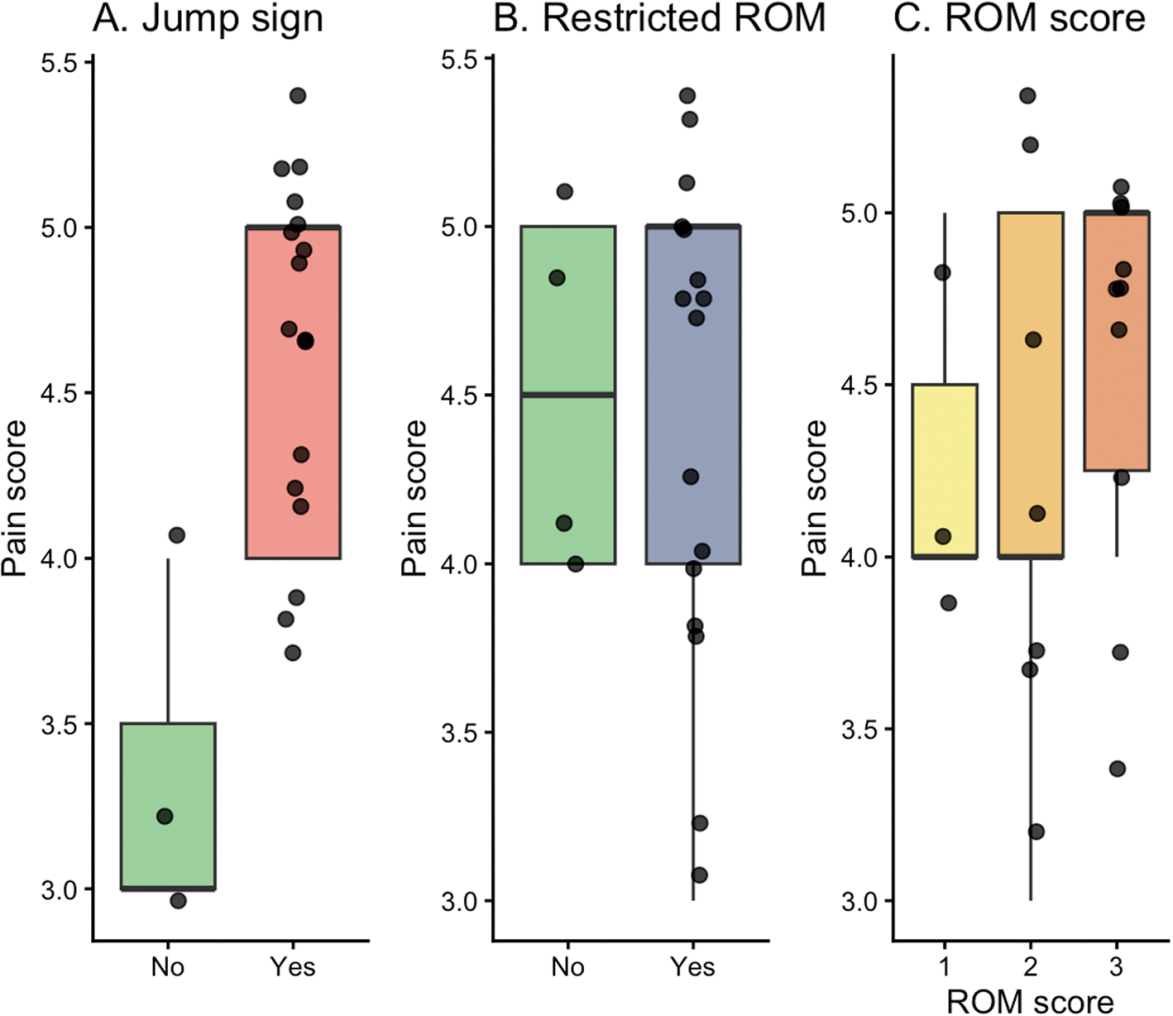
Pain score according to selected clinical variables. Pain scores are shown according to the presence or absence of jump sign (**A**) and restricted thoracolumbar range of motion (ROM) (**B**), and across categories of thoracolumbar ROM score (**C**). Boxplots represent the median and interquartile range, with whiskers indicating the range of observed values. Individual data points correspond to each horse. Higher values indicate greater pain

Clinical findings were not associated with the magnitude of objective gait asymmetries. Pain score and jump sign were not related to head or pelvic asymmetry measures in either unridden or ridden conditions, nor did they modify the effect of the rider. Restricted thoracolumbar ROM was not included in the models due to limited variability across horses. Thoracolumbar ROM score was explored descriptively as a clinical variable but did not show a consistent pattern in relation to asymmetry measures. The distribution of pain scores in relation to selected clinical variables is shown in Fig. 4.

Across all models, the inclusion of clinical variables did not materially improve model fit or explain variability in asymmetry outcomes. Estimates for these associations are presented in Table 4.

**Table 4:** Fixed-effects estimates for the association between clinical findings and absolute asymmetry measures

Outcome (absolute)	Model	Clinical variable	Estimate (β)	95% CI	*p*-value
HDmin	GLMM	Pain score	0.041	− 0.096 to 0.178	0.552
		Jump sign (yes/no)	0.063	− 0.141 to 0.267	0.541
PDmin	LM	Pain score	− 0.012	− 0.087 to 0.063	0.748
		Jump sign (yes/no)	0.021	− 0.094 to 0.136	0.721
HDmax	LM	Pain score	0.058	− 0.061 to 0.177	0.329
		Jump sign (yes/no)	0.074	− 0.108 to 0.256	0.418
PDmax	LM	Pain score	− 0.033	− 0.134 to 0.068	0.515
		Jump sign (yes/no)	0.052	− 0.097 to 0.201	0.489

Estimates represent the adjusted effect of each clinical variable on asymmetry magnitude. Positive estimates indicate increased asymmetry. All models were adjusted for rider condition, sex, age, body weight, and training level. A random intercept for horse was included when supported by between-horse variance (HDmin). Due to the limited number of horses in the intermediate and advanced training categories, estimates are presented descriptively. Confidence intervals correspond to Wald 95% intervals. IC95% calculated as Wald intervals (β ± 1.96·SE). Restricted thoracolumbar ROM was not included in the models owing to limited variability. A random intercept for horse was included when supported by between-horse variance (HDmin). Acronyms as in Table 1

### RHpE: descriptive results

**Fig. 5:**
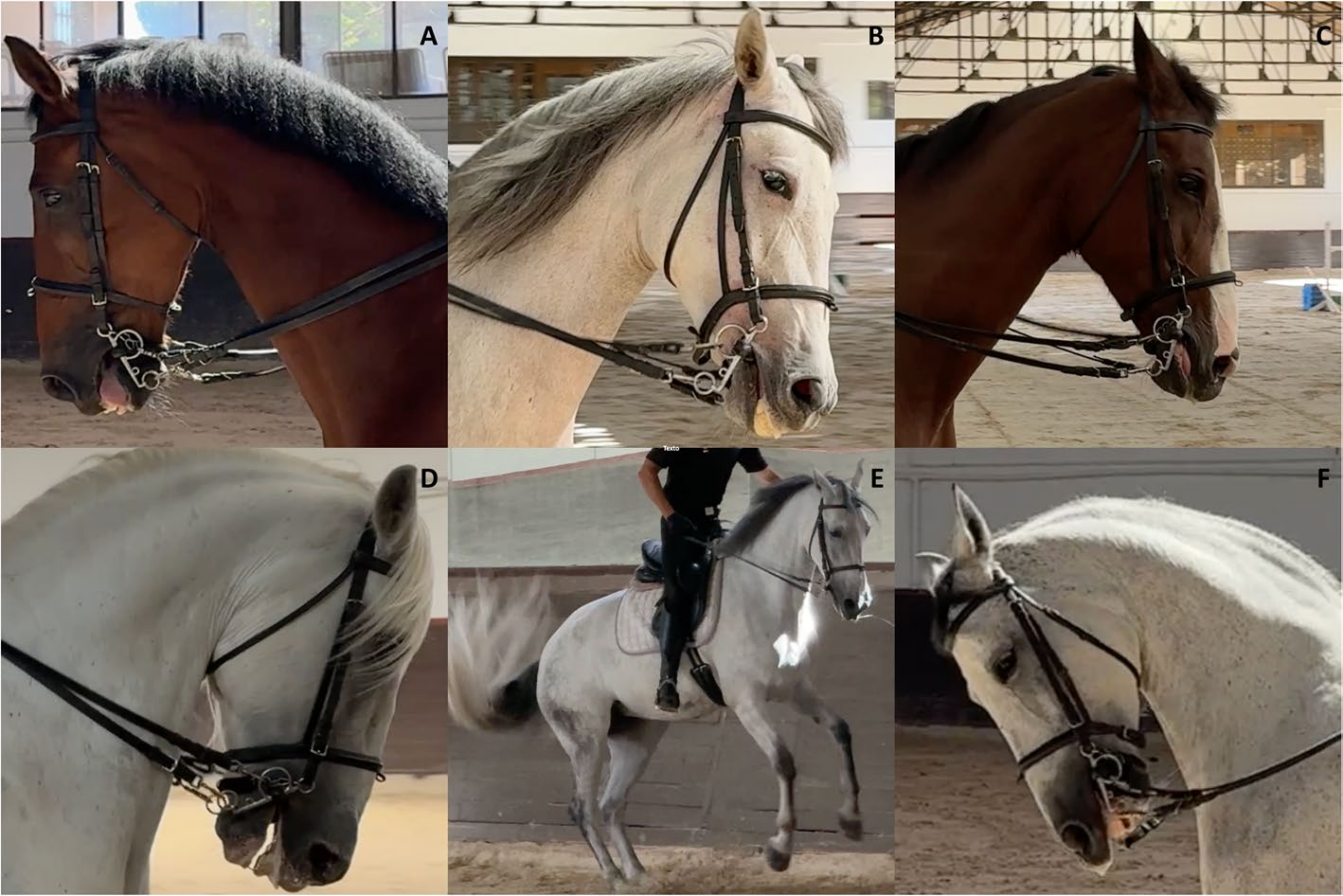
Pain-related behaviours during ridden work. **A** A large part of the tongue is protruding. Ears erect with the pinnae rotated outward. **B** Ears rotated outward, tension in the *Levator anguli oculi medialis* muscle, mouth open with teeth exposed. **C** Ears flat, intense stare with tension in the *Levator anguli oculi medialis* muscle*,* tongue protrusion. **D** Ears rotated outwards, head behind the vertical ≥ 10º, mouth open with complete separation of the teeth. **E** Rearing with both forelimbs off the ground, ears rotated outward, tension in the *Levator anguli oculi medialis* muscle*.* **F** Ears flat, head behind the vertical ≥ 10º and tilted, nose to the left

The execution time of the reprise averaged 8.5 min, with minimum and maximum values of 6 and 10 min, respectively. The mean RHpE score was 7.2 (SD 2.3; range 3–12). Eight of the 20 horses (40%) scored ≥ 8 behaviours, a threshold previously proposed as indicative of clinically relevant pain-related behaviour during ridden work. The most frequently observed pain-related behaviours were ears rotated back behind vertical (19/20; 95%), mouth opening (19/20; 95%), intense stare (16/20; 80%), sclera exposure (15/20; 75%), head tossed/twisted (14/20; 70%), and tail swishing (14/20; 70%). Less common behaviours included tail clamped (10/20; 50%), head behind vertical (9/20; 45%), hindlimb not tracking forelimb (8/20; 40%), and head tilted (5/20; 25%). Canter leg changes and bucking/kicking were each reported in 3 horses (15%), head up and down, tongue exposed, and spontaneous changes in gait were each observed in 2 horses (10%), and bit pulled, reluctance to move, and head in front of the vertical were each noted in 1 horse (5%). Eyes closed, rushed gait, gait too slow, toe dragging, change in direction, and rearing were not observed (0/20; 0%). Examples of some of these signs are presented in Fig. 5.

### Associations between RHpE, clinical findings, and ridden gait asymmetries

#### Correlations between RHpE and clinical variables

Spearman correlation analysis revealed that no associations between RHpE score, and clinical variables were statistically significant after adjustment for multiple comparisons using the false discovery rate (Benjamini–Hochberg method). Correlation coefficients were generally weak, and even the largest observed association (restricted ROM) did not reach statistical significance (Table 5).

**Table 5:** Spearman correlation coefficients between the Ridden Horse Pain Ethogram (RHpE) score and clinical variables

Variable	ρ (Spearman)	p-value	Adjusted p-value (FDR)	n
Jump sign	0.17	0.468	0.923	20
Restricted thoracolumbar ROM	− 0.33	0.157	0.627	20
Pain score	− 0.07	0.772	0.923	20
Thoracolumbar ROM score	− 0.02	0.923	0.923	20

Spearman’s rank correlation coefficients (ρ) are presented. P-values were adjusted for multiple comparisons using the false discovery rate (Benjamini–Hochberg method). No statistically significant associations were identified

#### RHpE and ridden gait asymmetries

Exploratory linear models including forelimb (HDmax, HDmin) and hindlimb (PDmax, PDmin) asymmetry measures did not meaningfully explain variation in RHpE scores. Neither head nor pelvic impact- or push-off–related asymmetry variables were associated with RHpE magnitude. The inclusion of pain score or training level as additional covariates did not substantially improve model fit, and overall explained variance remained low across all models. Estimates from exploratory regression analyses are summarised in Table 6.

**Table 6:** Exploratory linear regression models evaluating the association between the RHpE and variables assessed under the ridden condition

Predictor	Estimate (β)	95% CI	*p*-value
Model A. Clinical variables			
Pain score	0.86	− 0.90 to 2.61	0.329
ROM score	—	—	—
Model B. Forelimb biomechanical asymmetries			
HDmax	− 1.50	− 5.14 to 2.14	0.430
HDmin	− 0.91	− 3.09 to 1.27	0.425
Model C. Hindlimb biomechanical asymmetries			
PDmax	− 2.13	− 7.47 to 3.21	0.445
PDmin	− 2.20	− 9.48 to 5.08	0.562
Model D. Contextual variables			
Training level	− 0.86	− 2.25 to 0.53	0.223
Pain score	0.86	− 0.78 to 2.49	0.318

Estimates represent adjusted effects on RHpE score. None of the models showed meaningful explanatory power. All models were fitted using linear regression. Confidence intervals correspond to Wald 95% intervals. Thoracolumbar ROM score was constant across horses and could not be evaluated. Overall model fit was low across all models

## Discussion

The present study investigated the relationship between thoracolumbar MPS, objective gait asymmetries under unridden and ridden conditions, and pain-related behaviours during ridden work in police horses. Contrary to the initial hypothesis, thoracolumbar pain indicators assessed by palpation were not associated with objective gait asymmetries or RHpE scores.

### Clinical relevance of MPS indicators

The presence of hypersensitive spots associated with a taut band in 100% of the horses is consistent with human studies, where these criteria are considered essential for the diagnosis of MPS through palpation [10, 11].

The absence of LTRs in this region aligns with human findings, where LTRs in low back muscles have only been observed under ultrasound-guided TrPs injections [42]. In addition, LTRs have been shown to have poor reliability in humans and are considered confirmatory rather than essential for MPS diagnosis [11, 43]. Based on clinical experience, LTRs are detectable only during dry needling of the equine back muscles. Visualisation in this region is challenging due to the depth of the musculature and the flat-plane palpation technique. This observation could be consistent with human studies, where the pressure required to elicit a LTR varies by muscle [11].

The jump sign was considered clinically relevant and was detected in a high proportion of horses (85%). As its identification relies on facial and body expressions of pain in non-verbal animals, this finding aligns with equine studies where such expressions are essential for diagnosing musculoskeletal pain [15, 44, 45]. In particular, the jump sign has been used as an inclusion criterion for evaluating the brachiocephalicus muscle [13], and facial and behaviour pain-related expressions have been considered in the assessment of pectoral muscles [12]. This contrasts with human studies, where the jump sign is not regarded as an essential diagnostic criterion for MPS [10, 11]. Restricted ROM was the least observed sign (80%). These results are consistent with human studies, where it is not considered essential for MPS diagnosis [10, 11].

### Association between MPS indicators and gait asymmetries in unridden and ridden conditions

It is important to acknowledge that the gait analysis used in the present study, based on the Sleip system, quantifies vertical asymmetries of the head and pelvis and does not directly measure thoracolumbar kinematics such as lateral bending or axial rotation. Therefore, the gait variables used in this study should be interpreted as proxies of global vertical movement symmetry rather than as direct measures of spinal function.

In horses, kinematic analysis using motion capture systems have been used to evaluate back movement and to differentiate horses with and without back pain, demonstrating measurable differences in spinal kinematics between groups [28, 29]. However, the applicability of these technologies for assessing myofascial back pain in horses remains to be explored.

Evidence in humans relating myofascial low back pain to gait kinematics remains limited and has been explored across multiple planes; with very low-quality evidence [46]. However, our study measured vertical displacement asymmetry of the head and pelvis. Therefore, the findings are not directly comparable. Consequently, the absence of an association between clinical findings and the vertical gait symmetry measures in the present study cannot be readily interpreted based on current evidence.

Within the context of equine musculoskeletal pain, these results contrast with other studies [44, 47], which demonstrated that induced joint pain was associated with increased asymmetries. This discrepancy may be explained by the different nature and anatomical location of the pain models. In those studies, pain was induced in the tarsocrural joint, which would be expected to produce marked and measurable vertical displacement asymmetries. In contrast, in our study MPS was evaluated in the back. MPS is considered to produce a diffuse pain pattern with low-grade or subclinical lameness [4], with induced asymmetries that may be more subtle, variable, or below the detection threshold of vertical asymmetry metrics derived from head and pelvic motion. Another possible explanation for these differences is that they reflect acute experimentally pain versus more chronic naturally occurring pain conditions. In addition, it has been suggested that locomotor asymmetries are more pronounced in unilateral lameness compared with bilateral lameness, where compensatory mechanisms may reduce detectable asymmetries [48]. In this context, back pain may share similarities with bilateral lameness, as the primary source of pain is located close to the midline of the horse, potentially resulting in less apparent vertical displacement asymmetries.

The absence of an association between clinical signs and vertical gait asymmetry in either unridden or ridden conditions suggests that the influence of the ridden condition was largely independent of these clinical signs. Despite this, HDmax of the forelimb increased when horses were ridden, indicating that it may be particularly sensitive to the presence of a rider. However, the biomechanical interpretation of HDmax remains unclear, and its clinical relevance should be interpreted with caution. Compared with pelvic motion, head movement is more easily influenced by external factors such as rider input [49], and changes in neck posture. In trot, a raised neck position may increase reliance on active cervical musculature, particularly in horses working in a higher head carriage, which could influence forelimb mechanics [50]. Additionally, the forelimbs play a primary role in weight bearing and braking during ridden work, which may contribute to changes in head movement symmetry variables [50]. In contrast, previous work has reported no effect of sitting trot on vertical movement symmetry of the head and pelvis, suggesting that the symmetrical load induced by the rider does not substantially affect vertical symmetry variables [51]. This could be explained by differences in the experimental setting, as the present study was conducted outdoors with greater influence from uncontrolled external stimuli. Subtle rider asymmetries or minor, unintentional changes in seat and rein aids may also have contributed to the observed changes.

### Effect of training level on gait asymmetries under ridden conditions

In the present study, advanced trained horses showed greater increase forelimb push-off asymmetry. A possible explanation for these findings may involve individual horse-specific and training-related factors. In one advanced-level horse, a marked forelimb irregularity was observed that had not been clinically diagnosed as lameness. This horse had performed intensive work on hard ground during police exhibitions in the days preceding data collection, which could have contributed to a transient increase in gait asymmetry. For the remaining horses, the cause remains uncertain, although training-related postural adaptations may contribute. It has been described that high-trained dressage horses adopt a higher and more sustained head-neck posture during ridden exercise, which may reduce tension in the nuchal ligament and dorsal neck. Such effects could expose strength-related asymmetries, potentially contributing to larger push-off related asymmetries observed under ridden conditions [50]. However, these findings should be interpreted with caution, given the small number of horses at higher training levels.

### RHpE behavioural findings

Ears rotated behind vertical was the most frequently observed pain-related behaviour in this study. This finding aligns with previous studies in which ears position was considered a strong indicator of pain [17, 52–54]. Mouth opening was the second most frequently observed sign, as reported in previous studies [55]. It has been reported more commonly in lame horses, although it has also been documented in sound horses [17], and has been described as a conflict-related behaviour [56], which complicates its interpretation as a pain indicator. According to the rider, some horses appeared more comfortable after the experimental test following a change of bit, suggesting that this behaviour may be more consistent with a conflict-related origin. An intense stare was the third most frequently detected sign, consistent with previous studies describing it as one of the most significant indicators of pain [17, 53]. Additionally, mouth opening, an intense stare, and repeated tail swishing were the most frequent RHpE behaviours reported in elite dressage horses [57], which is consistent with the results of the current study.

### Association between MPS indicators, the RHpE, and gait asymmetries under ridden conditions

In this study, no significant correlation was found between MPS indicators and the RHpE. This finding aligns with a previous study reporting that the presence of thoracolumbar epaxial muscle pain or increased muscle tension did not influence the RHpE score [58]. This reinforces the idea that experienced palpation remains an essential method for diagnosing MPS, as evidenced in the human literature [59, 60], while the RHpE may help identify signs of musculoskeletal pain related to TrPs.

No significant association was found between ridden gait asymmetries and the RHpE in the present study. To the authors’ knowledge, no studies have been found that directly relate objectively measured gait asymmetries to the RHpE, limiting direct comparison with our results. However, previous research has reported associations between the RHpE, and gait asymmetries assessed subjectively, showing a significant relationship between the presence of lameness and higher RHpE scores [58]. This may be explained by methodological differences and by the exclusion of horses diagnosed with lameness not associated with MPS in the present study.

### Clinical and methodological implications of non-significant associations

The absence of significant associations between palpation-based MPS indicators and both gait asymmetries and ridden horse pain-related behaviours raises important questions regarding the functional relevance of clinically identified myofascial TrPs in the equine thoracolumbar region. These findings suggest that palpation-detected signs do not necessarily translate into measurable changes in locomotor symmetry or ridden behaviour.

One possible interpretation is that compensatory neuromuscular control strategies may allow horses to maintain apparently symmetrical movement patterns despite underlying nociceptive input, resulting in subtle biomechanical and behavioural changes that can fall below the detection threshold of current objective gait analysis.

From a methodological perspective, these findings highlight the limitations of relying on isolated outcome measures in the assessment of equine MPS. Clinically, this supports the concept that gait symmetry alone may be insufficient to fully characterise the functional expression of MPS. This reinforces the clinical value of experienced palpation as the primary tool for identifying MPS in horses, while gait analysis and behavioural scoring systems should be considered complementary methods that could help to contextualise functional expression. Accordingly, a multimodal assessment approach is required for diagnostic evaluation and clinical interpretation, consistent with recommendations in the human literature [61].

Overall, these results emphasise the need for further work to clarify the relationship between MPS and functional performance, and to identify outcome measures with higher sensitivity to clinically meaningful change in equine MPS.

### Strengths and limitations

The main strengths of this study include the involvement of an experienced veterinary clinician in MPS, specialised in TrPs assessment, which enhances palpation reliability [62], as well as the use of highly homogeneous conditions. The same experienced rider handled and rode all horses, and the same tack model, and terrain surfaces were used for all horses, reducing potential confounding factors. Trot was performed in sitting position to minimise asymmetries [51, 58], and a standardised reprise adapted to each horse’s work level was used to assess a full repertoire of movements, as recommended [63]. An additional strength was that RHpE scoring was performed from videos after a temporal separation from physical assessments, ensuring assessor blinding, while the rider was blinded to the palpation findings.

There are several limitations in this study. The absence of a control group is a relevant limitation, as it would have strengthened the interpretation of whether the observed associations were specific to horses with clinical features consistent with MPS. Future studies should therefore include an appropriate control group to better determine the specificity of these findings. Other limitations of the study include that the gait analysis was restricted to vertical head and pelvic movement. Therefore, subtle changes in back-specific motion potentially associated with MPS may not have been captured. Future research should extend the gait analysis to include additional kinematic parameters. All assessments were performed by a single trained observer. This approach improved consistency; however, it may have introduced systematic bias. Future studies should assess intra-rater reliability and include multiple trained assessors to evaluate inter-observer reliability and reduce the risk of systematic bias. Another limitation includes the relatively small sample size, which may have limited the generalisability of the findings. This relatively small sample size, inherent to field-based clinical studies conducted under operational conditions, should be considered when interpreting the results. Although the study was adequately sensitive to detect moderate-to-large within-horse effects, such as the observed change in HDmax between unridden and ridden conditions, its ability to identify small associations between clinical findings, gait asymmetries, and RHpE scores was limited. Therefore, the absence of statistically significant relationships for several variables should not be interpreted as definitive evidence of no effect, but rather as an indication that smaller or more variable effects may not have been detectable within this dataset. Future studies with larger sample sizes and more heterogeneous populations may help to further elucidate these relationships. A further limitation is that the camera could only be positioned in a fixed standard location, resulting in recordings captured from a single angle. Future work should consider multi-angle or multi-camera recording approaches to improve the capture of movement adaptation associated with back pain. No oral examination was conducted to identify potential lesions that could have influenced behaviour, and the tack was not assessed prior to the study by a qualified saddle fitter to identify possible equipment-related sources of pain. Future research should incorporate oral examinations and formal tack assessments to better account for alternative pain-related contributors. Finally, a wider range of equipment and riding contexts, including non-skilled riders, different surfaces, and varying training styles, should also be considered in future studies [64].

## Conclusion

This is the first study to explore the relationship between equine thoracolumbar MPS assessed by palpation, objectively measured gait asymmetries under unridden and ridden conditions, and pain-related behaviours during ridden work measured with the RHpE. In this population of working horses, MPS indicators were not associated with gait asymmetries or RHpE scores. However, the ridden condition, particularly in horses at higher training levels, influenced forelimb push-off asymmetry, suggesting that rider presence and training demands may affect specific biomechanical parameters independently of clinical findings. These findings support a multimodal approach that integrates clinical, biomechanical, and behavioural assessments to improve the diagnostic accuracy of MPS.

## Supplementary Information

Additional file 1. Individual data of the horses included in the study.

Additional file 2. Table 1. Palpation scoring scale used to evaluate the thoracolumbar region. Table 2. Mobility scoring scale used to evaluate the thoracolumbar range of motion (ROM).

Additional file 3. The dressage-type test performed by the 20 horses in this study. Time: 5-10 minutes.

Additional file 4. Summary of the 24 behaviours of the Ridden Horse Pain Ethogram (RHpE). A total behaviour score of 8 (out of 24) is likely to indicate the presence of musculoskeletal pain. s=seconds.

Additional file 5. Individual asymmetry values for each horse under in-hand and ridden conditions.

## Data Availability

The datasets used and/or analysed during the current study are available from the corresponding author on reasonable request.

## Abbreviations

MPS: Myofascial pain syndrome
TrP: Trigger point
LTR: Local twitch response
ROM: Range of motion
RHpE: Ridden horse pain ethogram
AI: Artificial intelligence
app: Smartphone application
HDmin: Forelimb impact asymmetry
HDmax: Forelimb push-off asymmetry
PDmin: Hindlimb impact asymmetry
PDmax: Hindlimb push-off asymmetry
LM: Linear model
GLMM: Generalized linear mixed-effects model
