# Supplementary material for: A multimodal approach to equine thoracolumbar myofascial pain: integrating clinical examination, AI-based gait analysis, and the ridden horse pain ethogram
Source: BMC Vet Res. 2026 Jun 9;22:474. doi: 10.1186/s12917-026-05566-w (PMC13474909; doi:10.1186/s12917-026-05566-w)
Supplement: Supplementary file 1 [file 12917_2026_5566_MOESM1_ESM.docx]

**Additional file 1**

Individual data of the horses included in the study

| Animal | Gender  (M/G) | Age (Years) | Weight  (Kg) | Breed | Discipline | Training level |
| --- | --- | --- | --- | --- | --- | --- |
| 1 | Mare | 9 | 560 | Warmblood | Dressage | Basic |
| 2 | Gelding | 13 | 511 | Spanish Pure Horse | Dressage | Basic |
| 3 | Gelding | 16 | 511 | Spanish Pure Horse | Dressage | Basic |
| 4 | Mare | 6 | 524 | Spanish Pure Horse | Dressage | Basic |
| 5 | Mare | 16 | 589 | Crossbreed | Dressage | Basic |
| 6 | Gelding | 14 | 511 | Spanish Pure Horse | Dressage | Advanced |
| 7 | Mare | 5 | 470 | Spanish Pure Horse | Dressage | Basic |
| 8 | Mare | 18 | 560 | Spanish Pure Horse | Dressage | Basic |
| 9 | Mare | 6 | 504 | Spanish Pure Horse | Dressage | Basic |
| 10 | Mare | 10 | 553 | Warmblood | Showjumping | Intermediate |
| 11 | Mare | 16 | 524 | Spanish Pure Horse | Dressage | Advanced |
| 12 | Mare | 9 | 490 | Crossbreed | Dressage | Advanced |
| 13 | Gelding | 10 | 464 | Warmblood | Showjumping | Intermediate |
| 14 | Mare | 6 | 470 | Crossbreed | Showjumping | Basic |
| 15 | Mare | 7 | 498 | Spanish Pure Horse | Dressage | Basic |
| 16 | Mare | 12 | 538 | Spanish Pure Horse | Dressage | Basic |
| 17 | Gelding | 18 | 504 | Crossbreed | Dressage | Basic |
| 18 | Gelding | 15 | 458 | Spanish Pure Horse | Dressage | Basic |
| 19 | Mare | 5 | 511 | Spanish Pure Horse | Dressage | Basic |
| 20 | Mare | 6 | 532 | Warmblood | Showjumping | Basic |
