# Supplementary material for: A multimodal approach to equine thoracolumbar myofascial pain: integrating clinical examination, AI-based gait analysis, and the ridden horse pain ethogram
Source: BMC Vet Res. 2026 Jun 9;22:474. doi: 10.1186/s12917-026-05566-w (PMC13474909; doi:10.1186/s12917-026-05566-w)
Supplement: Supplementary file 2 [file 12917_2026_5566_MOESM2_ESM.docx]

**Additional file 2**

**Table 1.** Palpation scoring scale used to evaluate the thoracolumbar region in this study.

| **Score** | **Description** |
| --- | --- |
| 0 | Soft, low tone |
| 1 | Normal |
| 2 | Increased muscle tone but not painful |
| 3 | Increased muscle tone and/or painful (slight associated spasm on palpation, no associated movement) |
| 4 | Painful (associated spasm on palpation with associated local movement, i.e., pelvic tilt, extension response) |
| 5 | Very painful (spasm plus behavioural response to palpation, i.e., ears flat back, kicking) |

**Table 2.** Mobility scoring scale used to evaluate the thoracolumbar range of motion (ROM).

| Score | Classification | Description |
| --- | --- | --- |
| 0 | Hypermobility | Increase in expected range of motion and less than normal resistance to movement. |
| 1 | Normal | Normal range of motion, expected movement, horse is supple. |
| 2 | Mild | Slight decrease in range of motion and minimal resistance to movement at the end of the range of motion when the part is moved. |
| 3 | Moderate | Significant decrease in expected range of motion and significant resistance to movement. |
| 4 | Severe | No range of motion, this part is rigid and stiff. |
