# Supplementary material for: A multimodal approach to equine thoracolumbar myofascial pain: integrating clinical examination, AI-based gait analysis, and the ridden horse pain ethogram
Source: BMC Vet Res. 2026 Jun 9;22:474. doi: 10.1186/s12917-026-05566-w (PMC13474909; doi:10.1186/s12917-026-05566-w)
Supplement: Supplementary file 3 [file 12917_2026_5566_MOESM3_ESM.docx]

**Additional file 3**

The dressage-type test performed by the 20 horses in this study. Time: 5-10 minutes.

| Exercise | References | Description |
| --- | --- | --- |
| 1 | H | Enter in walk. Track to the right. |
|  | C | Down the centre line. |
|  | G | Figure of eight starting to the left. |
| 2 | M | Shoulder-in right (basic).  Haunches-in to the right on the second half of the long side (intermediate and advanced). |
| 3 | B | Half circle in leg-yield (basic) or in half-pass to the right (intermediate and advanced). Track to the left. |
| 4 | R | Allow the reins slightly, and medium walk to C. |
| 5 | C | Down the quarter line. Extended walk. |
| 6 | EXB | At E, turn and track across the arena. At B, collected walk. |
| 7 | B | Shoulder-in left (basic)  Haunches-in to the left on the second half of the long side (intermediate and advanced). |
| 8 | M | Half circle in leg-yield (basic) or in half-pass to the left (intermediate and advanced). |
| 9 | BXE | At B, turn and track across the arena. At X, transition to trot. |
| 10 | XC | Three-loop serpentine between the quarter lines with transitions to halt and rein back on the centre line (two transitions). |
| 11 | C | Down the centre line. Figure of eight starting to the left in working trot. |
| 12 | M | Shoulder-in right. |
| 13 | B | Half circle in leg-yield (basic) or in half pass to the right (intermediate and advanced). |
| 14 | M | Collected trot.  Down the quarter line. Medium trot to E. |
|  | C |  |
| 15 | EXB | At E, turn and track across the arena in working trot. At B, shoulder-in left. |
| 16 | M | Half circle in leg-yield (basic) or in half-pass to the left (intermediate and advanced). |
| 17 | BX | At B, turn across the arena. At X, transition to canter right.  Depending on the horse’s reaction, ride a 15 m circle or continue. |
| 18 | C | 15 m circle.  At B, turn and track across the arena. At X, transition to trot. |
|  | BX |  |
| 19 | EIM | Diagonal in medium trot. |
| 20 | M | Transition to left canter.  Depending on the horse’s reaction, ride a 15 m circle or continue. |
|  | C |  |
| 21 | EX | Turn across the arena. At X, ride a 15 m circle and transition to trot. |
| 22 | BIH | Diagonal in medium trot. At H, transition to walk. |
| 23 | HC | Walk on a long rein and leave the arena. |
