# Supplementary material for: A multimodal approach to equine thoracolumbar myofascial pain: integrating clinical examination, AI-based gait analysis, and the ridden horse pain ethogram
Source: BMC Vet Res. 2026 Jun 9;22:474. doi: 10.1186/s12917-026-05566-w (PMC13474909; doi:10.1186/s12917-026-05566-w)
Supplement: Supplementary file 4 [file 12917_2026_5566_MOESM4_ESM.docx]

**Additional file 4**

Summary of the 24 behaviours of the Ridden Horse Pain Ethogram. A total behaviour score of 8 (out of 24) is likely to indicate the presence of musculoskeletal pain. s=seconds.

**Behaviour description**

1. Repeated changes in head position up and down, not in rhythm with the trot.

2. Head tilted repeatedly

3. Head in front of vertical (>30◦) for ≥10 s

4. Head behind vertical (>10◦) for ≥10 s

5. Repeated changes in head position from side to side

6. Ears rotated back behind vertical (both or one only) for ≥5 s; repeatedly lay flat

7. Eye lids closed or half closed for 2–5 s

8. Sclera exposed repeatedly

9. Intense stare (glazed expression, ‘zoned out’) for ≥5 s

10. Mouth opening ± shutting repeatedly with separation of teeth for ≥10 s

11. Tongue exposed, protruding or hanging out and/or moving in and out repeatedly

12. Bit pulled through the mouth on one side (left or right) repeatedly

13. Tail clamped tightly to middle or held to one side

14. Tail swishing: large movements, repeatedly up and down/side to side/circular; repeatedly during transitions

15. A rushed gait (frequency of trot steps >40/15 s); irregular rhythm in trot or canter; repeated changes of speed in trot or canter

16. Gait too slow (frequency of trot steps <35/15 s); passage-like trot

17. Hindlimbs do not follow tracks of forelimbs but repeatedly deviated to left or right; on 3 tracks during trot or canter

18. Canter repeated incorrect strike off with wrong forelimb leading; repeated change in leg in front and/or behind (disunited or cross-cantering)

19. Spontaneous changes in gait (e.g., breaks from canter to trot or trot to canter)

20. Stumbles or trips more than once in front or behind; repeated bilateral hindlimb toe drag

21. Sudden change in direction against rider’s cues; spooking

22. Reluctance to move forwards (has to be kicked ± verbal encouragement); stops spontaneously

23. Rearing (both forelimbs off the ground)

24. Bucking or kicking backwards (one or both hindlimbs)
