# Supplementary material for: A multimodal approach to equine thoracolumbar myofascial pain: integrating clinical examination, AI-based gait analysis, and the ridden horse pain ethogram
Source: BMC Vet Res. 2026 Jun 9;22:474. doi: 10.1186/s12917-026-05566-w (PMC13474909; doi:10.1186/s12917-026-05566-w)
Supplement: Supplementary file 5 [file 12917_2026_5566_MOESM5_ESM.docx]

**Additional file 5**

Individual asymmetry values for each horse under in-hand and ridden conditions

| Horse | HDmin in hand | HDmin ridden | HDmax in hand | HDmax ridden | PDmin in hand | PDmin ridden | PDmax in hand | PDmax ridden |
| --- | --- | --- | --- | --- | --- | --- | --- | --- |
| 1 | 0.31754214 | 0.04427380 | 0.41819929 | 0.56757248 | 0.06327427 | 0.02324317 | 0.17462469 | 0.135427742 |
| 2 | 0.01929095 | 0.64639417 | 0.01886184 | 0.21947767 | 0.19940075 | 0.23671570 | 0.20907346 | 0.354341973 |
| 3 | 0.41229535 | 0.20063780 | 0.04527835 | 0.23220765 | 0.57207189 | 0.24357877 | 0.02302688 | 0.334135284 |
| 4 | 0.33620700 | 0.07123305 | 0.14010193 | 0.01004944 | 0.02098638 | 0.26500649 | 0.21939502 | 0.137682572 |
| 5 | 0.61897813 | 0.58605487 | 0.45140349 | 0.60195175 | 0.20241546 | 0.09756020 | 0.12824991 | 0.102760728 |
| 6 | 0.15319705 | 0.07881362 | 0.48568771 | 0.71900402 | 0.44709245 | 0.25408297 | 0.33560523 | 0.111271077 |
| 7 | 0.51796515 | 1.54933609 | 0.09805406 | 1.15453335 | 0.05036961 | 0.52702822 | 0.88463285 | 0.465114544 |
| 8 | 0.16910957 | 0.16660374 | 0.10386582 | 0.61060643 | 0.27663895 | 0.09021460 | 0.18532818 | 0.204000080 |
| 9 | 0.10486575 | 0.26029492 | 0.53722104 | 0.80796197 | 0.09266019 | 0.04150723 | 0.79737065 | 0.329985076 |
| 10 | 0.20416610 | 1.36780938 | 0.09670573 | 0.41804037 | 0.45366359 | 0.24428542 | 0.15313713 | 0.003648821 |
| 11 | 0.05685664 | 1.37759214 | 0.20987878 | 0.36586169 | 0.24378260 | 0.05139273 | 0.36144021 | 0.036041022 |
| 12 | 0.03686204 | 0.48589404 | 0.05455975 | 0.44044652 | 0.47215736 | 0.50759364 | 0.35304487 | 0.367894285 |
| 13 | 0.31523056 | 0.27296859 | 0.30317626 | 1.03562486 | 0.18054311 | 0.35485758 | 0.41540158 | 0.223271734 |
| 14 | 0.23786764 | 0.12368885 | 0.09010444 | 0.49783289 | 0.15691848 | 0.26943883 | 1.15331628 | 0.676887223 |
| 15 | 0.51982037 | 0.90693141 | 0.27692881 | 0.32292826 | 0.07773755 | 0.11354077 | 0.22367525 | 0.568203115 |
| 16 | 0.08752981 | 0.22470852 | 0.01467088 | 0.84510149 | 0.40635434 | 0.10489412 | 0.31399271 | 0.201139885 |
| 17 | 0.23635184 | 0.49639598 | 0.23161554 | 0.24076842 | 0.12850839 | 0.17785695 | 0.24601654 | 0.028178719 |
| 18 | 0.40318558 | 0.05741577 | 0.16240354 | 0.34197030 | 0.07999110 | 0.14537738 | 0.06560457 | 0.576820270 |
| 19 | 0.05933112 | 0.01779337 | 0.02076827 | 0.55160798 | 0.21327260 | 0.25221999 | 0.13210375 | 0.022475408 |
| 20 | 0.54432354 | 0.46954070 | 0.27928164 | 0.44314782 | 0.58238433 | 0.41301060 | 0.33213108 | 0.225916185 |

Values represent absolute asymmetry measures derived from the Sleip system. Each horse was evaluated under both in hand and ridden conditions. Values closer to zero indicate greater symmetry. HDmin, head impact mean; HDmax, head push-off mean; PDmin, hind impact mean; PDmax, hind push-off mean
